# High‐Dimensional Variable Selection With Competing Events Using Cooperative Penalized Regression

**DOI:** 10.1002/bimj.70036

**Published:** 2025-02-18

**Authors:** Lukas Burk, Andreas Bender, Marvin N. Wright

**Affiliations:** ^1^ Leibniz Institute for Prevention Research and Epidemiology ‐ BIPS Bremen Germany; ^2^ Department of Statistics Ludwig‐Maximilians‐Universität München München Germany; ^3^ Faculty of Mathematics and Computer Science University of Bremen Bremen Germany; ^4^ Munich Center for Machine Learning (MCML) Munchen Germany; ^5^ Department of Public Health University of Copenhagen Kobenhavn Denmark

**Keywords:** competing risks, Cox regression, high‐dimensional data analysis, penalized regression, variable selection

## Abstract

Variable selection is an important step in the analysis of high‐dimensional data, yet there are limited options for survival outcomes in the presence of competing risks. Commonly employed penalized Cox regression considers each event type separately through cause‐specific models, neglecting possibly shared information between them. We adapt the feature‐weighted elastic net (fwelnet), an elastic net generalization, to survival outcomes and competing risks. For two causes, our proposed algorithm fits two alternating cause‐specific models, where each model receives the coefficient vector of the complementary model as prior information. We dub this “cooperative penalized regression,” as it enables the modeling of competing risk data with cause‐specific models while accounting for shared effects between causes. Coefficients that are shrunken toward zero in the model for the first cause will receive larger penalization weights in the model for the second cause and vice versa. Through multiple iterations, this process ensures stronger penalization of uninformative predictors in both models. We demonstrate our method's variable selection capabilities on simulated genomics data and apply it to bladder cancer microarray data. We evaluate selection performance using the positive predictive value for the correct selection of informative features and the false positive rate for the selection of uninformative variables. The benchmark compares results with cause‐specific penalized Cox regression, random survival forests, and likelihood‐boosted Cox regression. Results indicate that our approach is more effective at selecting informative features and removing uninformative features. In settings without shared effects, variable selection performance is similar to cause‐specific penalized Cox regression.

## 1 Introduction

High‐dimensional data such as gene expression and other omics settings continue to grow in importance for clinical applications. In that regard, variable selection methods are a fundamental part of many analytic procedures that aim to identify the most relevant components of ever‐growing datasets. Variable selection in the field of survival analysis can be complicated by the presence of competing events, which are commonly modeled using cause‐specific hazards models. These approaches fit a model for each event type by treating events of the competing risk as censored observations, which allows the application of common techniques such as Cox regression to the competing risks setting. By combining all cause‐specific models, one can fully represent the data‐generating process (Beyersmann et al. 2009).

One disadvantage of this approach is that learning covariate effects on low prevalence event types can have low power. In some scenarios, however, two competing events may share certain underlying physiological mechanisms, for example, age affects the probability for dying of different causes. If covariates can be of predictive value for both competing events rather than only one, there is a need for variable selection approaches that can utilize information about effects shared between events, especially if one of them only occurs rarely.

We introduce a new method for variable selection in the competing risks setting that is based on the popular Elastic Net (Zou and Hastie 2005; Simon et al. 2011) and the Feature‐Weighted Elastic Net (Tay et al. 2023). Our method iteratively fits two cause‐specific penalized Cox regression (Coxnet) models where the penalization weights are influenced by the complementary model's estimated coefficients, leading to lower penalization weights for covariates with large effects for the other cause. This adjustment ensures that covariates of relevance for either event are less likely to be removed from the model. Analogously, larger penalization weights are successively amplified, therefore increasing the chance of removing noise variables from the model. Effects shared between the two competing events are therefore more likely to be taken into account, leading to the inclusion of coefficients that might otherwise be shrunken to zero, or alternatively removing otherwise spurious effects. As this method builds upon the familiar elastic net, we present it as an extension for use in high‐dimensional settings under aforementioned assumptions, where we expect it to yield improved variable selection results. Conversely, without the presence of a shared effect, our method is expected to reduce to a set of conventional Coxnet models. Since this procedure enables two cause‐specific models influencing each other iteratively, we dub this procedure “Cooperative Penalized Regression” (CooPeR).

Previous works in the area of elastic net extensions include the Random Approximate Elastic Net (RAEN) introduced by Sun and Wang (2023), which uses a subdistribution hazards approach and aims to address stability concerns with variable selection methods in high‐dimensional settings. The Repeated Elastic Net (RENT) is an ensemble method introduced by Jenul et al. (2021), which similarly emphasizes stability, yet does not consider the survival setting. The Priority Lasso by Klau et al. (2018) extends the LASSO with an approach to utilize block‐wise priorities to apply different penalization weights to covariates belonging to different blocks. Ambrogi and Scheike (2016), on the other hand, propose an alternative approach to competing risk modeling with penalization, based on binomial regression.

In Section 3.1, we demonstrate our method in a low‐dimensional setting, where we expect that information sharing through our approach reduces bias in coefficient estimates. We then compare CooPeR with three established methods often applied in high‐dimensional survival settings (Section 3.2): penalized Cox regression, random survival forests (RSFs), and CoxBoost (Binder et al. 2009; Ishwaran et al. 2008), in a simulation study based on previous work by Binder et al. (2009), emulating gene expression data. Variable selection performance of the four methods is evaluated based on the positive predictive value (PPV) and false‐positive rate (FPR) to investigate their ability to correctly select informative values and their susceptibility to the selection of uninformative (noise) variables. Finally, in Section 4, we apply CooPeR in a real data setting, identifying multiple shared effects that were not discovered by other methods.

## 2 Methods

### 2.1 The Feature‐Weighted Elastic Net

Penalized regression methods such as the LASSO and ridge regression are popular due to their ability to handle high‐dimensional data problems and perform variable selection in case of the former. The elastic net combines the two methods, and has been particularly popular as it allows a trade‐off between the variable selection of the $\ell_{1}$ (LASSO) and the $\ell_{2}$ (ridge) penalties. Since we build upon the elastic net, we first consider the elastic net objective function for linear regression as given by Zou and Hastie (2005):

(1) $$\begin{array}{cc} J(\beta ) & =\frac{1}{2}||y-{X\beta ||}_{2}^{2}+\lambda \sum_{j=1}^{p}\left(\alpha |\beta_{j}|+\frac{1-\alpha }{2}\beta_{j}^{2}\right), \end{array}$$

where $y\in R^{n}$ and $X\in R^{n\times p}$ represent the observed outcome and covariate matrix, respectively. We assume the covariates to be centered to omit $\beta_{0}$, such that $\beta \in R^{p}$ is the coefficient vector. λ≥0 is the sparsity parameter and α∈[0,1] controls the relative weight of the two penalty terms, where α=0 corresponds to a pure $\ell_{2}$‐penalty, and α=1 corresponds to a pure $\ell_{1}$‐penalty. For the purposes of variable selection, we are primarily concerned with the $\ell_{1}$ penalty, as only this will shrink coefficients to 0. The right component of the sum comprises the penalty, where λ applies to all coefficients $\beta_{j}$ equally.

In some scenarios, it may be desirable to adjust the penalization weights for individual features or groups of features, which would allow for more fine‐grained control of the variable selection process compared to the standard approach where all covariates are treated equally. Since it would not be feasible to define one $\lambda_{j}$ within the penalization term to fit penalization terms specific to covariates, additional structure needs to be imposed.

One approach is presented by the feature‐weighted elastic net (fwelnet), an extension of the elastic net that incorporates a weighting scheme to take prior information on covariate relevance or group structure into account (Tay et al. 2023). By assigning lower weights to features that are likely to be of greater relevance to the task, the corresponding coefficients are less likely to be shrunken to zero. Conversely, higher weights can increase the penalization weight, increasing the likelihood of uninformative covariates to be removed from the model. In addition, it allows to group covariates in different blocks, which is useful for high‐dimensional settings where, for example, clinical and gene expression or other omics data are combined. The latter application is related to existing approaches to grouped penalization, such as the group LASSO (Yuan and Lin 2006) or the Priority‐LASSO (Klau et al. 2018).

The fwelnet uses an information matrix $Z\in R^{p\times G}$, for p covariates and G denoting the number of sources of information for a grouped setting. $z_{j}^{T}\in R^{G}$ denotes the jth row. In the context of this work, a second application is more relevant: Z can be used to apply predetermined weights of features. Consider, for example, a regression setting with five covariates $x_{1},\text{…},x_{5}$, which are assumed to be of descending relevance for the outcome y, then a matrix a matrix $Z_{\text{ex}}\in R^{5\times 1}$ constructed as such:

(2) $$\begin{array}{c} Z_{\text{ex}}=\left(\begin{array}{c} 4\\ 2\\ 1\\ 0.5\\ 0.1 \end{array}\right) \end{array}$$

will influence the penalization weights such that covariates associated with larger values will receive smaller penalization weights and vice versa. This effect is achieved through the weight function $w_{j}\left(\theta \right)$ introduced in the fwelnet objective function:

(3) $$\begin{array}{cc} J(\beta_{0},\beta ) & =\frac{1}{2}||y-{X\beta ||}_{2}^{2}+\lambda \sum_{j=1}^{p}w_{j}\left(\theta \right)\left(\alpha |\beta_{j}|+\frac{1-\alpha }{2}\beta_{j}^{2}\right), \end{array}$$

where $\theta ={\left(\theta_{1},\text{…},\theta_{G}\right)}^{⊤}$ is a $R^{G}$ hyperparameter that needs to be selected. The score $z_{j}^{⊤}\theta$ can be thought of as an indicator for how influential feature j is on the response, while $\theta_{g},\;g=1,\text{…},G$ represents how important the gth source of feature information is in identifying which features are important. The computation of the objective is accomplished through an algorithm that alternates between the optimization of β and θ (see Tay et al. 2023, Section 3.1).

The weight function $w_{j}\left(\theta \right)$ was chosen by Tay et al. (2023) as

(4) $$w_{j}\left(\theta \right)=\frac{\sum_{l=1}^{p}\exp\left(z_{l}^{T}\theta \right)}{p\exp(z_{j}^{T}\theta )},$$

largely motivated by its useful properties, such as collapsing to the original elastic net penalty for θ=0 and not resulting in negligible penalty factors for large z. Due to this transformation, the prior weights set in Z do not directly translate to penalization weights, but merely determine a relative weight for the covariates. This allows the interpretation of $z_{j}$ encoding a relative importance of features, which is then expressed directly in the optimization problem. It should be noted that due to the form of $w_{j}\left(\theta \right)$, entries in z are informative due to their relative magnitudes, whereas values such as $z_{j}=1$ do not necessarily result in a penalization weight of $w_{j}\left(\theta \right)=1$, or otherwise imply some notion of neutrality. One can then assign appropriate values for covariates one has reason to assume are more or less relevant for the given task. This can be particularly useful to ensure that a coefficient that is expected to be small but associated with a relevant covariate nonetheless is not shrunken to 0 and thereby removed from the model.

The use case for vector‐valued Z has been explored further by Tay et al. (2023) in a simulation setting where Z is set to a noisy version of the absolute coefficients |β| of the data‐generating model. This corresponds to Z containing imprecise prior information about the relative importance of features, and resulted in the improved predictive performance in said simulation settings. This idea then motivates the multitask algorithm the authors introduce to make use of this property. This algorithm uses the coefficient vector of an initial penalized regression model as prior information matrices $Z_{1}$ and $Z_{2}$ for a task with two distinct targets $y_{1}$ and $y_{2}$ and shared X. By iteratively fitting new models using the previous iteration's coefficient estimates as prior information, the algorithm amplifies the relative covariate effects and can therefore more effectively remove uninformative covariates, or analogously preserve informative ones. In the next subsection, we will use the idea of this multitask algorithm and extend it to survival settings, to improve variable selection in the presence of competing risks.

### 2.2 Cooperative Penalized Regression

As the original feature‐weighted elastic‐net was formulated in the generalized linear model framework, we first need to extend it to survival outcomes. We assume right‐censored event times $T_{i}=\min\left(Y_{i},C_{i}\right),\Delta_{i}=I\left(T_{i}\leq C_{i}\right)$ with observations $(t_{i},\delta_{i},x_{i}),\;i=1,\text{…},n$. We are interested in modeling the survival function S(t) via the hazard function h(t),

(5) $$\begin{array}{cc} S(t) & =P(T>t)=1-F(t), \end{array}$$

(6) $$\begin{array}{cc} h(t) & =\frac{f(t)}{S(t)}=\lim_{\Delta t\to 0}\frac{P(t<T\leq t+\Delta t\;|\;T>t)}{\Delta t}, \end{array}$$

h(t) is commonly modeled with Cox regression, where the hazard rate

(7) $$\begin{array}{c} h\left(t|x_{i}\right)=h_{0}\left(t\right)\exp\left(\beta^{T}x_{i}\right) \end{array}$$

is defined by the regression coefficient vector β and $h_{0}$ represents the arbitrary baseline hazard.

We adapted the reference implementation provided by Tay et al. (2023) to allow survival outcomes by minimization of the negative Cox partial likelihood similar to existing implementations (see Simon et al. 2011). The objective function 3 is thereby extended to Cox regression:

(8) $$\begin{array}{cc} \left(\hat{\beta },\hat{\theta }\right) & =\underset{\beta ,\theta }{\mathrm{argmin}}\sum_{i=1}^{n}\mathrm{NLL}\left(t_{i},\delta_{i},x_{i}^{T}\beta \right)\\ & \;+\lambda \sum_{j=1}^{p}w_{j}\left(\theta \right)\left[\alpha |\beta_{j}|+\frac{1-\alpha }{2}\beta_{j}^{2}\right], \end{array}$$

with $w_{j}\left(\theta \right)$ identical to Equation (4) and NLL referring to the negative log‐likelihood term derived from the Cox partial likelihood:

(9) $$L\left(\beta \right)=\prod_{j=1}^{r}\frac{\exp(\beta^{⊤}x_{\left(j\right)})}{\sum_{l\in R(t_{\left(j\right)})}\exp\beta^{T}x_{l}},$$

where $t_{\left(1\right)}<\text{…}<t_{\left(r\right)}$ are assumed to be ordered survival times without ties, and $R(t_{\left(j\right)})$ is the set of observations at risk at time $t_{\left(j\right)}$.

With this adaption, single‐event survival data can be modeled analogously to existing implementations. We refer to this as “fwcoxnet” to distinguish it from the original formulation.

In the presence of competing risks, the event indicator $\delta_{i}$ can take values k∈{0,1,…,K} for one of K competing events. As we focus on cause‐specific methods, we retain the notation of k∈{1,2} for two distinct event types or causes, but in the following, we use $\delta_{k}$ to denote the binary event indicator where 0 indicates censoring and 1 indicates occurrence of event k, and $\beta_{k}$ denotes the coefficient vector for the model specific to event k.

For our approach to competing event modeling, we now combine two elements: The first consists of a simulation setting explored by Tay et al. (2023) in which the feature information matrix Z is set to be a noisy version of the true coefficient vector |β|. The authors show that fwelnet can utilize even this noisy information to improve test Mean Squared Error (MSE) in their simulation study. A similar effect can be expected in a survival setting.

The second element is the multitask algorithm referred to in Section 2.1, which describes an iterative approach where two distinct outcomes $y_{1}$ and $y_{2}$ are modeled using the same dataset X by making use of the “noisy information” approach highlighted by the first element. We adapt this algorithm to the competing risk survival setting by substituting $y_{k}$ with $\left(t_{k},\delta_{k}\right)$ to denote the cause‐specific event times t and binary event indicator δ, using the fwcoxnet extension for survival outcomes. The resulting Algorithm 1 models two competing events simultaneously.

ALGORITHM 1 **The fwelnet for competing risks: Cooperative penalized regression (CooPeR).**

**Table:** 

1. Initialize ${\hat{\beta }}_{1}^{\left(0\right)}$ and ${\hat{\beta }}_{2}^{\left(0\right)}$ at the lambda.min elastic net solutions for $\left(X,t_{1},\delta_{1}\right)$ and $\left(X,t_{2},\delta_{2}\right)$ respectively, i.e. at the λ which maximizes the cross‐validated log‐likelihood. 2. For j=0,1,… until a stopping criterion is reached a. Fit $\mathrm{fwcoxnet}\left(X,t_{2},\delta_{2},Z_{2}=\left|\beta_{1}^{\left(j\right)}\right|\right)$ to determine ${\hat{\beta }}_{2}^{\left(j+1\right)}$. b. Fit $\mathrm{fwcoxnet}\left(X,t_{1},\delta_{1},Z_{1}=\left|\beta_{2}^{\left(j+1\right)}\right|\right)$ to determine ${\hat{\beta }}_{1}^{\left(j+1\right)}$.

Similarly to the previously introduced multitask algorithm, this approach uses the estimated coefficients of two penalized regression models as the prior information for individual fwcoxnet fits. A stopping criterion for Algorithm 1 could be the root mean square difference of consecutively estimated coefficient vectors, $\left|\right|\beta_{k}^{\left(j\right)}-\beta_{k}^{\left(j-1\right)}{\left|\right|}_{2}<\varepsilon$, for, for example, $\varepsilon =10^{-8}$.

Assuming that a subset of covariates in X has predictive value for both competing events, their corresponding coefficients are less likely to be estimated to 0 in either cause‐specific model. Due to the alternating nature of the algorithm, this then leads to, for example, $x_{j}$ receiving a larger penalization weight relative to other covariates in the cause‐specific model for event 1 if the model for event 2 produced $\beta_{2j}=0$, where index 2j refers to the jth covariate in the model specific to cause 2.

The method is implemented in the cooper R package available at https://github.com/jemus42/cooper , which is based on the original implementation of the *fwelnet* algorithm Tay et al. (2023).

## 3 Simulation Studies

We evaluate CooPeR in two distinct simulation settings. The first experiment in Section 3.1 focuses on a low‐dimension setting where we investigate the coefficient estimation behavior throughout iterations of Algorithm 1. The second setting in Section 3.2 evaluates the variable selection performance in a high‐dimensional setting based on previous work in this area (Binder et al. 2009).

### 3.1 Proof of Concept

We conduct a small‐scale simulation study to investigate the general behavior of our approach in a low‐dimensional, high‐signal setting. For this purpose, we simulated competing risk data with N=1000 observations and p=14 features across four scenarios based on a piecewise‐exponential hazard model (Bender and Scheipl 2018; Beyersmann et al. 2009):

A: $X_{1}$ has equal effect of 1 on both cause‐specific hazards, with both causes having equal proportion of approximately 35%. We expect CooPeR to perform well in this scenario as large $\left|\hat{\beta_{1}}\right|$ leads to a smaller penalization weight for $\left|\hat{\beta_{2}}\right|$ and vice versa, hence allowing for mutual amplification.
B: $X_{1}$ has an effect of 1 on the cause 1 hazard only, $X_{2}$ has an equal effect on the cause 2 hazard 2 only, both causes having equal prevalence as in A. In this scenario, we expect CooPeR to not perform any differently than Coxnet, as there is no mutual information to share between causes.
C: $X_{1}$ has an effect of 1 on the cause 1 hazard and a smaller effect of 0.25 on the cause 2 hazard, with cause 1 having a higher prevalence (approximately 55%) than cause 2 (approximately 7%). CooPeR should be able to use the effect of $X_{1}$ on cause 1 to amplify its effect on the rarer cause 2, hence improving the coefficient estimate there.
D: $X_{1,2,3}$ have equal effects of 1, 0.75, and −0.5, respectively, on both cause‐specific hazards, with cause 2 being as prevalent as in setting C. Our expectations here were similar to C, with the addition of multiple, smaller effects.

In each setting, $X_{1,2,3}$ are uniformly distributed random variables in [−3,3] and 11 additional variables drawn from a multivariate standard normal distribution without any effect on either event type. CooPeR is run for five iterations (mt_max_iter = 5), a convergence threshold of thresh = 1e‐7, and an initial learning rate of t = 100. Both thresh and t are control parameters of fwelnet, and are not specific to CooPeR. In general, we would expect CooPeR to show reduced coefficient biases compared to Coxnet in settings A and C, whereas in settings B and D, we primarily expect CooPeR and Coxnet to show comparable results.

**FIGURE 1:**
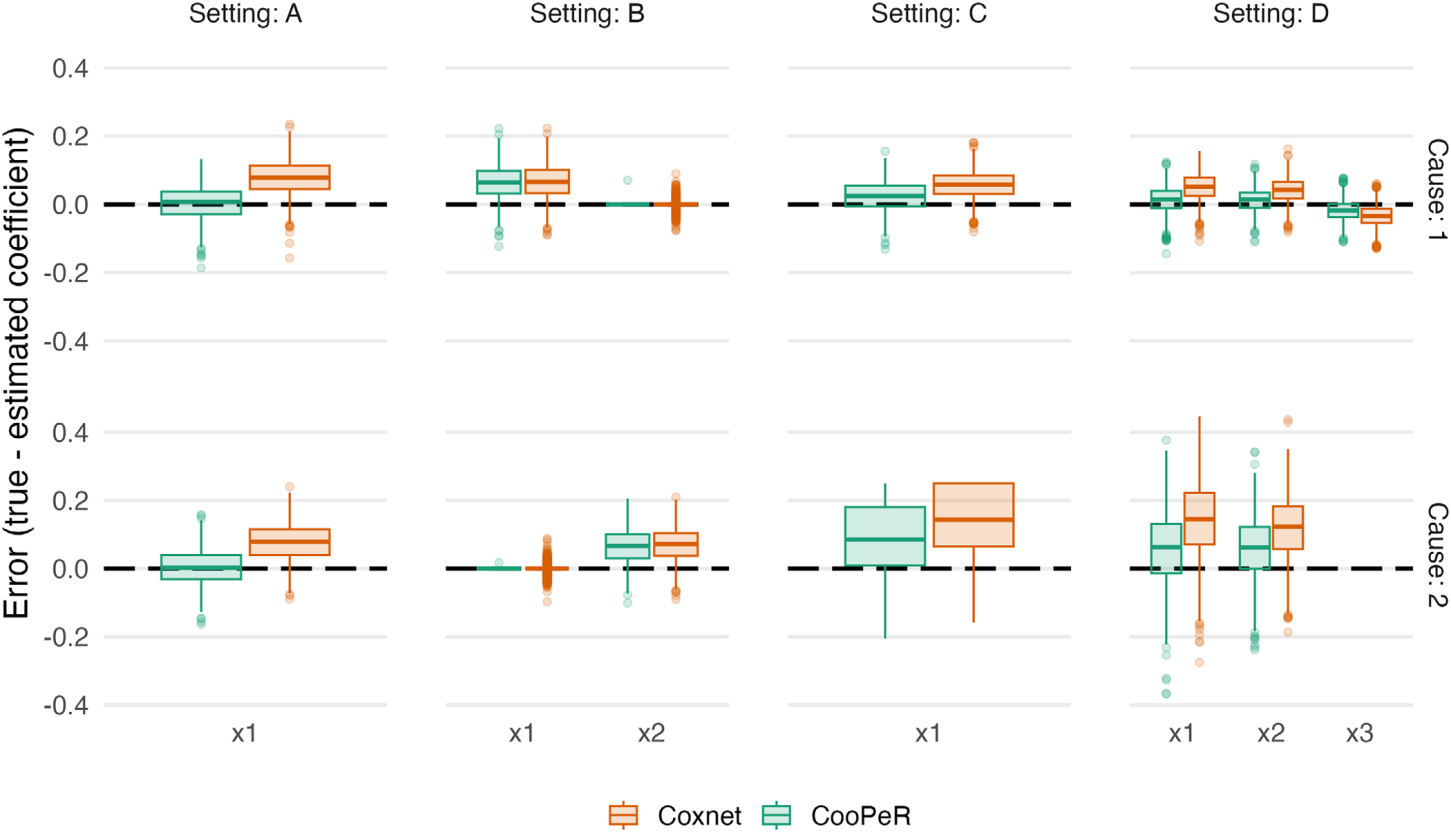
Coefficient estimation bias $\beta_{j}-{\hat{\beta }}_{j}$ for CooPeR and Coxnet across 1000 simulation replicates. Each column shows one simulation setting, and each row is specific to one cause‐specific model. Settings A, C, and D have varying degrees of mutual information for CooPeR to exploit, leading to noticeably reduced bias ($\beta_{j}-{\hat{\beta }}_{j}$) in the coefficient estimates. Setting B shows CooPeR yielding very similar results to the Coxnet.

We investigate the error in the coefficient estimation, that is, $\beta_{j}-{\hat{\beta }}_{j}$ associated with $X_{j}$ for CooPeR and Coxnet, respectively. Results displayed in Figure 1 largely meet expectations across all settings, as CooPeR either exhibits a greatly reduced estimation bias, or largely equivalent results compared to Coxnet. In setting A, we observe nearly unbiased coefficient estimates for $X_{1}$ in both cause 1 and cause 2, indicating that the present mutual information in fact lead to a “debiasing” of the coefficient estimate. In setting B, CooPeR and Coxnet produce very similar results as was expected, with the exception of slightly reduced variability for CooPeR as indicated by a smaller number of outliers. In settings C and D, we do not observe the same near‐unbiased estimates as in setting A, yet CooPeR still produces improved coefficient estimates compared to Coxnet in terms of bias. Particularly, the smaller effects in setting D show reduced errors across both causes. All in all CooPeR's coefficient estimates are either similar to Coxnet at the worst, or almost unbiased in the case where strong shared effects are present.

### 3.2 High‐Dimensional Data

We employ a simulation setup based on the experiment conducted by Binder et al. (2009), which is described in more detail by Binder and Schumacher (2008a). This setup allows to investigate the variable selection performance in the presence of shared information, opposing effects, and no shared information between two event types. The data‐generating process is as follows: p=5000 covariates are generated for n=400 observations drawn from a univariate standard normal distribution N(0,1) and transformed to achieve the desired block‐correlation structure. The covariate blocks are assigned as follows:

1. Block 1: (“Mutual”) 250 covariates with correlation of ρ≈0.5 and 4 informative covariates with increasing effect on both hazards.
2. Block 2: (“Reversed”) 250 covariates with correlation of ρ≈0.35 and four informative covariates with increasing effect on the first cause hazard and decreasing effect on the second cause hazard.
3. Block 3: (“Disjoint 1 & 2”) 500 covariates with correlation of ρ≈0.05 and eight informative covariates. These are further divided into subblock 3.1 with 4 covariates having a decreasing effect on the cause 1 hazard, and subblock 3.2 with four covariates having an increasing effect on the cause 2 hazard only.
4. Block 4: (“Cor. Noise”) 500 noninformative covariates with correlation of ρ≈0.32 without effect on either hazard.
5. Noise: The remaining 3500 covariates are uncorrelated and have no effect on either hazard.

Effects $\beta_{j}$ are assigned as −0.5 or 0.5 for a decreasing and increasing effect on the event‐specific hazards, respectively. Survival and censoring times are generated following Bender, Augustin, and Blettner (2005):

$$\begin{array}{cc} T_{i,k} & =-\frac{U_{i,k}}{\lambda \cdot \exp(x_{i}^{T}\beta_{k})},\;i=1,\cdots ,n,\;k\in \left\{1,2\right\}\\ C_{i} & =-\frac{U_{C_{i}}}{\lambda_{C}}, \end{array}$$

where $U_{i,k}$ and $U_{C_{i}}$ are standard uniform random variables, $\lambda =\lambda_{C}=0.1$ is the baseline hazard of cause k, $x_{i}^{T}$ is the vector of observation i, and $\beta_{k}$ is the vector of coefficients for cause k. This process results in approximately 30% probability for event 1, 40% for event 2, and 30% for censoring.

The following methods were used for comparison:

#### CooPeR

The algorithm was executed with parameters mt_max_iter = 3, alpha = 1, t = 100, and thresh = 1e‐7. Setting alpha = 1 corresponds to the α parameter of the elastic net and applies the $\ell_{1}$ for variable selection. The remaining parameters ensure that the algorithm is more likely to find an optimal solution for the internal θ parameter, and mt_max_iter = 3 is set to limit the number of iterations of Algorithm 1. λ is determined through 10‐fold cross‐validation within each iteration.

#### Coxnet

The initial step of the *CooPeR* procedure is equivalent to a penalized cause‐specific Cox regression fit as described in Algorithm 1. The method is implemented in the *glmnet* R package and extends the elastic net to Cox regression (Friedman, Tibshirani, and Hastie 2010; Simon et al. 2011). It is used as the primary baseline of comparison for *CooPeR*, with hyperparameter alpha = 1, and λ determined through 10‐fold cross‐validation.

#### CoxBoost

A gradient‐boosting approach specific to survival models implemented in the *CoxBoost* R package, described in Binder and Schumacher (2008b) and Binder et al. (2009). This method estimates cause‐specific coefficient vectors, which were interpreted analogously to *CooPeR* and *Coxnet* results. We set cmprsk = "csh", while parameters penalty and stepno are tuned using the packages' ‘optimCoxBoostPenalty()‘ function.

#### RSF

RSF is an extension of the popular random forest algorithm to survival data (Breiman 2001; Ishwaran et al. 2008), which has been further extended to competing risk settings (Ishwaran et al. 2014). We apply the implementation provided by the *randomForestSRC* R package (Ishwaran and Kogalur 2023) to fit cause‐specific models. For variable selection, the resulting out‐of‐bag variable importance vectors v for each cause‐specific model are classified as informative if $v_{j}>\left|\min v\right|$ for j∈1,⋯,p. This approach is based on a technique introduced by Janitza, Celik, and Boulesteix (2018) and similar to the “Vita” method Degenhardt, Seifert, and Szymczak (2019). We tune mtry and nodesize using the packages’ tune() function.

Models are evaluated across 1000 replications of the simulation process. For classification purposes, each covariate block is considered separately for each cause, that is, the number of covariates per block serves as the denominator for the calculation of the true positive counts and related measures. True positives are defined as informative covariates that are selected by the method via a nonzero coefficient or variable importance estimate, and false positives are defined as noninformative covariates that are selected by the method. Other classification metrics are derived analogously, with the primary evaluation metrics being PPV ($\frac{\mathrm{TP}}{\mathrm{TP}+\mathrm{FP}}$) and FPR ($\frac{\mathrm{FP}}{\mathrm{FP}+\mathrm{TN}}$). As individual covariate blocks may not contain any true effects for a given cause, the PPV is not defined in these cases. This affects Block 3.1 for cause 2, Block 3.2 for cause 1, and Block 4 for both causes. In these scenarios, only FPR is calculated.

The different methods are evaluated in terms of their ability to detect true effects and to remove noise variables within each of the respective blocks. For additional evaluation metrics such as the true positive rate (TPR, sensitivity, $\frac{\mathrm{TP}}{P}$) and the $F_{1}$ score, which combines PPV and TPR, see Appendix A. In addition to the variable selection performance, we also assess predictive performance by fitting cause‐specific Cox models using the selected variables produced by each model (see Appendix A). In this procedure, the Cox model serves as a neutral method to focus on the differences in the quality of the selected variables, rather than model‐specific predictive ability, as this is the focus of this work. Based on the time‐dependent AUC and Brier scores (Gerds and Kattan 2021), we see consistently better predictive performance of the models based on variables selected by CooPeR than models based on variables selected by the other methods.

#### 3.2.1 Detection of Informative Variables (PPV)

**FIGURE 2:**
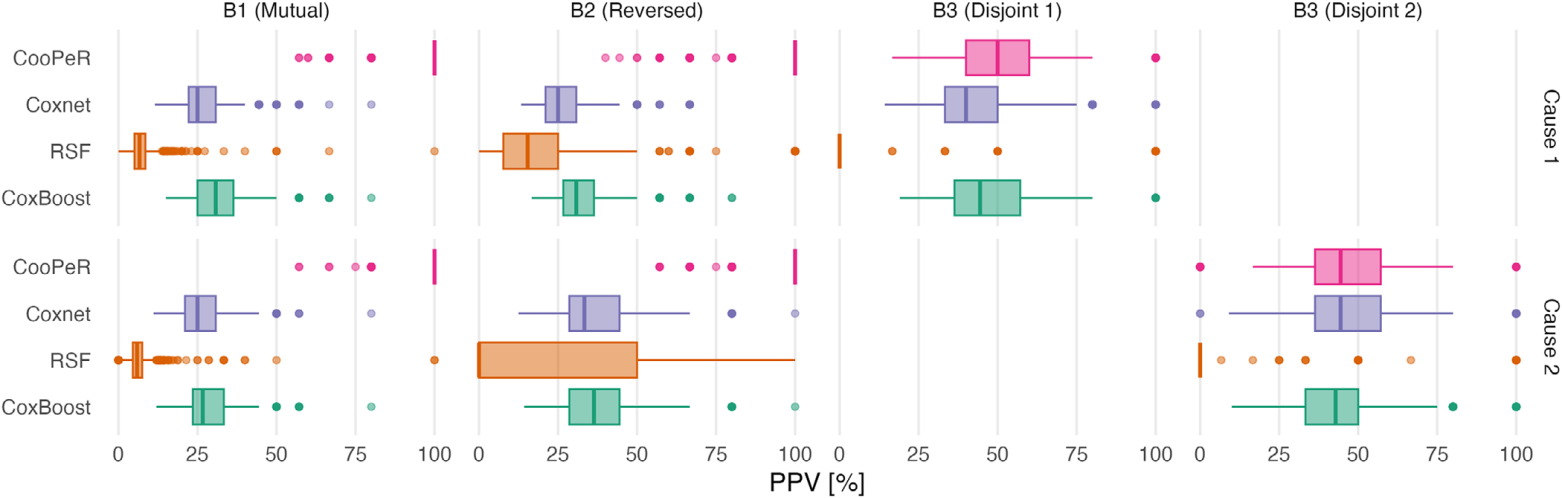
Positive predictive values of CooPeR, Coxnet, RSF, and CoxBoost across 1000 simulation runs. Columns B1–B3 correspond to covariate blocks with different effect structures. CooPeR (top row) shows very high PPV in blocks 1 and 2 where it is expected to perform well due to the presence of shared effects, whereas in block 3, we observe results not meaningfully different than those of the penalized Cox regression. Since block B3 contains two subblocks with covariates only informative for one cause respectively, there are no true‐positive covariates to select for subblock 2 and cause 1 and vice versa.

As PPV relies on the presence of true effects (informative variables) within a given covariate block, we only measure it in blocks 1–3, excluding subblocks 3.1 or 3.2, respectively, depending on which event of interest is considered. Figure 2 displays PPVs as horizontal boxplots across 100 simulation runs, with one column per covariate block and one row for causes 1 and 2, respectively. Results are additionally listed as median and IQR in Table A.1. Across all covariate blocks, the resulting scores do not differ meaningfully between cause 1 and cause 2.

CooPeR (top row) yields PPVs conclusively higher than the penalized Cox regression, RSF, and CoxBoost approaches in blocks 1 and 2. In these scenarios, CooPeR can leverage the shared information between both causes regardless of their direction, as only their magnitude is of relevance. The median PPV in these cases is 100% with a 25% quantile around 75%, which greatly outperforms the remaining methods’ median scores between 25% and 50%. Since we expect these blocks to show favorable results for our method, we consider the conclusive advantage over the reference methods to be a mark in favor of our approach.

Similarly, results in block 3 indicate CooPeR to perform not noticeably worse than the penalized Cox regression, which confirms our assumption that CooPeR will, at worst, behave similarly to the underlying Cox regression. RSF achieves very low PPV here with a median PPV of 0%, which is the result of a low number of true positive selections (often 0, rarely 1), while simultaneously yielding more false positives (up to 5) and resulting in a PPV of 0 in the majority of cases. CoxBoost, on the other hand, achieves by far the best scores in block 3 with median PPV of 100%.

#### 3.2.2 Susceptibility to Noise Variables (FPR)

**FIGURE 3:**
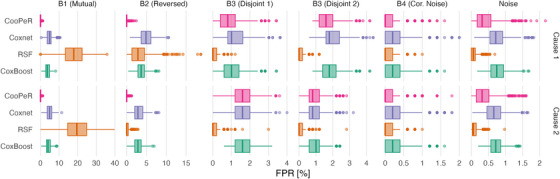
False positive rates of CooPeR, Coxnet, RSF, and CoxBoost across 1000 simulation runs for the different covariate blocks with different effect structures (B1–B4) and the block of pure noise variables. Similar to Figure 2, CooPeR performs well in blocks 1 and 2 with very low‐median FPR. In block 3, it performs similar to Coxnet, and slightly better in block 4 and the block of remaining noise variables. Note the varying *x*‐axis scaling introduced to accommodate widely differing FPR ranges.

The FPR indicates a method's propensity for falsely selecting uninformative variables, and therefore can be measured in all covariate blocks as each contain some amount of noise variables. FPR scores are displayed in Figure 3 as boxplots analogously to Figure 2. Additionally refer to Table A.2 for median and IQR FPR scores. Across all covariate blocks, the resulting scores do not differ meaningfully between cause 1 and cause 2 as was the case for the PPV results.

Blocks 1 and 2 again show favorable results for CooPeR, indicating that the algorithm is very effective at the removal of uninformative variables. CooPeR shows the lowest median FPR of 0% with little variability (IQR of 0.41%), meaning that it very consistently did not falsely select any noise variables.

Coxnet shows slightly higher FPR around 5%, occasionally falsely selecting dozens of noise variables. RSF shows uncharacteristically high FPR in block 1, whereas its FPR in block 2 is more in line with the other methods, but with higher variability. CoxBoost shows results similar to Coxnet in blocks 1 and 2.

In blocks 3.1 and 3.2, the disjoint effects do not greatly affect CooPeR's scores compared to the Coxnet reference, but both RSF and CoxBoost perform well here with FPRs below 0.5% compared to CooPeR's 1–3% range. RSF in particular yielding a median FPR of 0% in blocks 3 and 4. All methods achieve a median FPR of 0% in block 4 aside from Coxnet, with only marginally higher median of 0.2%, with the primary difference being the slightly reduced variability for RSF and CoxBoost.

In the covariate blocks consisting of uncorrelated noise variables only, all methods ranked similarly as in the previous blocks, with an overall lower FPR range and CooPeR yielding comparable results to Coxnet, while RSF and CoxBoost yield lower scores once again.

## 4 Application Example

Comparing the variable selection performance on a real‐world dataset is complicated by the lack of labels to indicate which variables hold true predictive power and which do not. To showcase CooPeR on a real‐world dataset, we make use of the bladder cancer survival dataset previously used in related literature (Binder et al. (2009); Dyrskjøt et al. (2005)). This dataset contains 301 observations with 192 censored, 74 experiencing death from bladder cancer (event 1), and 33 experiencing death from other causes (event 2). We apply CooPeR to this dataset in the following code snippet using the cooper function and setting parameters similar to those in the previous simulation study. Notably, we use the logical parameter stratify_by_status and nfolds to instruct cooper to subsequently use stratified fivefold cross‐validation for the internal λ optimization rather than the default 10‐fold cross‐validation that is not stratified by the event indicator (status). This ensures numerical stability, as datasets with few observations and rare events can lead to computational issues as the calculation of the Cox likelihood is complicated by small samples. We then extract the estimated event‐specific coefficients for both CooPeR and Coxnet using a standard coef method, where we access the Coxnet coefficients using the initial fit of Algorithm 1 with an aptly named logical argument use_initial_fit.


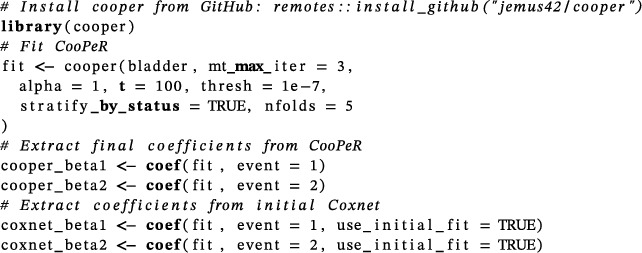




Based on this example, we see that the selected variables shared between the cause‐specific models differ for CooPeR and Coxnet. Coxnet identifies age as the only variable shared between the two causes, whereas CooPeR additionally identifies the microarray features "SEQ1176", "SEQ1226", "SEQ213", "SEQ250", "SEQ34", and "SEQ780". Of these, "SEQ34" is also identified by Binder et al. (2009) as a potential shared effect between the two causes, while "SEQ213" is also listed in the progression signature by Dyrskjøt et al. (2005).

We additionally applied RSF and CoxBoost to the same data and tuning both methods analogously to previous experiments, and found Coxnet and CoxBoost to identify "age" to be the only covariate effect shared between both causes, while RSF did not identify any shared effects. This indicates that CooPeR is more likely to identify shared effects than any of the other methods we considered in this setting. We assess prediction performance similarly to previous results based on cause‐specific Cox models using variables selected by each method in Section A.2, showing overall very similar performance of CooPeR, Coxnet, and CoxBoost, with RSF achieving slightly worse results.

## 5 Discussion

We have proposed a novel variable selection method based on the elastic net, referred to as CooPeR, and it performed well in simulation experiments. Our experiment shows improved variable selection capabilities compared to other methods, which is indicated by close to perfect PPVs and near zero FPRs in the presence of shared effects, meaning covariates with effects on both cause‐specific hazards. In these cases, CooPeR leverages this mutual information to more reliably remove noise variables and include informative variables compared to Coxnet, RSF, and CoxBoost, sometimes with a wide margin as seen in Figures 2 and 3.

The presence of this kind of mutual information is not an easily verifiable assumption in real‐word applications, so it is generally not straight‐forward to determine whether CooPeR can be expected to yield superior results compared to other methods. However, based on simulation results, CooPeR tends to perform similar to Coxnet without mutual information present. This comes with the caveat of additional computational overhead due to the additional optimization procedure of the internal parameter θ, which is performed in addition to the cross‐validation used internally to determine the optimal λ value. This means that, in the worst case, CooPeR would be both slower and less numerically stable than an equivalent Coxnet. In Section 4, we demonstrated CooPeR's ease of use in a real‐data example, where it was the only method that identified shared effects in the range of microarray features. However, since CooPeR is attuned to identifying shared effects specifically, it is not surprising to see it identifying more covariates here than other methods.

There remain multiple opportunities for further research with regard to the proposed algorithm. For one, the algorithm starts by fitting a model for cause 2, then using the coefficient estimate to inform the model fit on cause 1. This means that the order in which the event types are defined has a direct effect on the algorithm, as changing cause 1 to be cause 2 and vice versa would reverse the order of the model fits and therefore the initial order in which information flows. This effect might be negligible over multiple iterations, but in our experiments, we find that the procedure does not necessarily benefit from more than two or three iterations. In many real‐world applications, it is usually the case that event type 1 is the primary event of interest, for example, death from a specific disease, whereas event type 2 represents an aggregated event encompassing death from any other cause. In that case, it may be perfectly acceptable to attempt to extract additional information from the secondary cause‐specific model to inform the model fit on the primary event of interest. Related to the order of events is the question of the number of events. While it is possible to fit cause‐specific models for k>2 events, the proposed algorithm does not trivially generalize to even the k=3 case. In that scenario, one would need to define a rule by which the initial solutions $\beta_{1,2,3}^{\left(0\right)}$ should be used to define which feature information vector $Z_{1,2,3}^{\left(0\right)}$ and in which order. In lieu of a natural solution, empirical evidence may provide further insight into this scenario. Alternatively, the issue may be circumvented altogether by defining cause 1 to be the cause of interest, and aggregating all remaining causes under an umbrella cause 2 “any other causes.” While this process will lose a certain amount of detail, this is an often necessary practice as there are often few observed events for rarer causes. Additionally, Tay et al. (2023) note that the motivation for their implementation of the internal optimization routine of the θ parameter is motivated by computational constraints arising when K, the number of groups in Z, is large. For CooPeR, $Z\in R^{p\times 1}$ as there is no grouping by design. This should allow for a more efficient implementation for a scalar parameter. In our experiments, we found it necessary to allow the optimization process a sufficiently large computational budget by starting with a large learning rate t while setting a small threshold parameter thresh to not prematurely end the algorithm.

In summary, we have proposed a useful tool for variable selection in the challenging combination of high dimensionality and the presence of competing risks.

## Conflicts of Interest

The authors have declared no conflict of interest.

### Open Research Badges

This article has earned an Open Data badge for making publicly available the digitally‐shareable data necessary to reproduce the reported results. The data is available in the Supporting Information section.

This article has earned an open data badge “**Reproducible Research**” for making publicly available the code necessary to reproduce the reported results. The results reported in this article could fully be reproduced.

## Supporting information

Supporting Information

## Figures and Tables

**FIGURE A.1:**
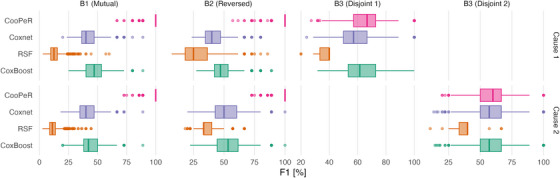
F1 score of CooPeR, Coxnet, RSF, and CoxBoost across 1000 simulation runs. Columns B1–B3 correspond to covariate blocks with different effect structures. CooPeR (top row) shows very high F1 in blocks 1 and 2 where it is expected to perform well due to the presence of shared effects, whereas in block 3, we observe results not meaningfully different than those of the penalized Cox regression, similar to the PPV scores.

**FIGURE A.2:**
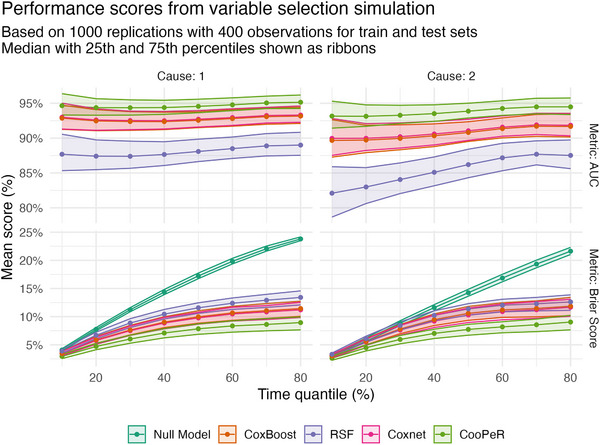
Prediction performance scores based on an independent test set of 400 observations of the simulated data as evaluated by the time‐dependent Brier scores and AUC across 10% quantiles of the test set. The Null model is added for reference for the Brier scores for reference only. Ribbons indicated the 25% and 75% quantile of the performance score at each time point across the 1000 simulation replications.

**FIGURE A.3:**
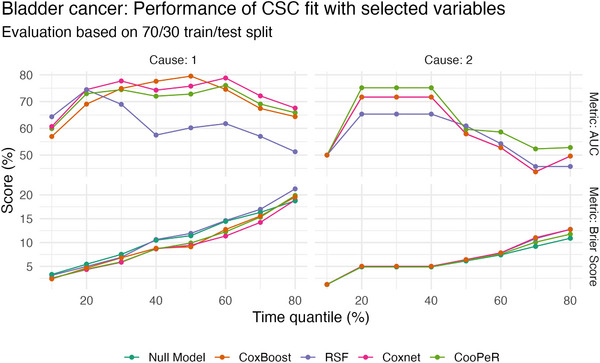
Prediction performance scores based on a 70/30 train‐test split of the bladder cancer dataset as evaluated by the time‐dependent Brier scores and AUC across 10% quantiles of the test set. The Null model is added for reference for the Brier scores for reference only. Scores are based on cause‐specific Cox models fit using the variables selected by each of the methods.

## Data Availability

Data sharing not applicable to this article as no datasets were generated or analyzed during the current study.

## Appendix A Additional Results

### A.1 Simulation Study: High‐Dimensional Data

Tables A.1 and A.2 contain the results shown in Section 3.2 in tabular form, and Table A.3 shows additionally the TPR (sensitivity). Since TPR is calculated as $\frac{\mathrm{TP}}{P}$, where P refers to the total number of positives (informative variables), and there are only four informative variables per covariate block, this measure can only take one of five values (0, 0.25, 0.5, 0.75, 1) for each method. We therefore find it to be only of limited use for evaluation in this context, and prefer the PPV as a measure.

**TABLE A.1:** Median (IQR) of PPV scores (%) across different covariate blocks for causes 1 and 2.

Block	CooPeR	Coxnet	CoxBoost	RSF
**Cause 1**				
B1 (Mutual)	**100 (0)**	25 (8.55)	30.77 (11.36)	6.67 (3.43)
B2 (Reversed)	**100 (0)**	25 (9.72)	30.77 (9.7)	15.38 (17.31)
B3 (Disjoint 1)	**50 (20)**	**40 (16.67)**	44.44 (20.78)	0 (0)
**Cause 2**				
B1 (Mutual)	**100 (0)**	25 (9.72)	26.67 (9.8)	5.88 (2.95)
B2 (Reversed)	**100 (0)**	33.33 (15.87)	36.36 (15.87)	0 (50)
B3 (Disjoint 2)	**44.44 (20.78)**	**44.44 (20.78)**	42.86 (16.67)	0 (0)

*Bold:* Method with best scores within each cause/block

**TABLE A.2:** Median (IQR) of FPR scores (%) across different covariate blocks for causes 1 and 2.

Block	CooPeR	Coxnet	CoxBoost	RSF
**Cause 1**				
B1 (Mutual)	0 (0)	4.88 (2.03)	3.66 (2.03)	**17.89 (8.94)**
B2 (Reversed)	0 (0)	**4.88 (2.44)**	3.66 (1.63)	2.85 (3.15)
B3 (Disjoint 1)	0.81 (0.81)	**1.01 (0.81)**	**1.01 (0.81)**	0 (0.2)
B3 (Disjoint 2)	1.6 (0.8)	**1.8 (0.8)**	**1.8 (0.8)**	0 (0.2)
B4 (Cor. Noise)	0 (0.2)	**0.2 (0.4)**	**0.2 (0.4)**	0 (0.2)
Noise	0.31 (0.4)	0.71 (0.37)	**0.74 (0.37)**	0.06 (0.11)
**Cause 2**				
B1 (Mutual)	0 (0)	4.88 (2.44)	4.07 (2.03)	**19.51 (10.16)**
B2 (Reversed)	0 (0)	**2.85 (2.03)**	**2.85 (1.63)**	0 (0.41)
B3 (Disjoint 1)	**1.6 (0.8)**	**1.6 (0.8)**	**1.6 (0.8)**	0 (0.2)
B3 (Disjoint 2)	0.81 (0.6)	0.81 (0.6)	**1.01 (0.6)**	0 (0.2)
B4 (Cor. Noise)	0 (0.2)	**0.2 (0.4)**	**0.2 (0.4)**	0 (0.2)
Noise	0.31 (0.37)	0.66 (0.4)	**0.71 (0.29)**	0.06 (0.11)

*Bold:* Method with best scores within each cause/block

**TABLE A.3:** Median (IQR) of TPR scores (%) across different covariate blocks for causes 1 and 2.

Block	CooPeR	Coxnet	CoxBoost	RSF
**Cause 1**				
B1 (Mutual)	**100 (0)**	**100 (0)**	**100 (0)**	75 (25)
B2 (Reversed)	**100 (0)**	**100 (0)**	**100 (0)**	25 (25)
B3 (Disjoint 1)	**100 (0)**	**100 (0)**	**100 (0)**	0 (0)
**Cause 2**				
B1 (Mutual)	**100 (0)**	**100 (0)**	**100 (0)**	75 (50)
B2 (Reversed)	**100 (0)**	**100 (0)**	**100 (0)**	0 (0)
B3 (Disjoint 2)	**100 (25)**	**100 (25)**	**100 (25)**	0 (0)

*Bold:* Method with best scores within each cause/block

Figure A.1 additionally shows the F1 scores of the models analogous to the figures presented in Section 3.2. The F1 score is calculated as the harmonic mean of TPR and PPV and can be calculated as $F_{1}=\frac{2\mathrm{TP}}{2\mathrm{TP}+\mathrm{FP}+\mathrm{FN}}$. Here, CooPeR shows very good performance in blocks 1 and 2, analogously to previous results for the PPV scores.

Figure A.2 shows the predictive performance of the variable selection methods presented in Section 3.2 and Figures 2 and 3. Scores are based on cause‐specific Cox models fit using the variables selected by each of the methods to form a neutral comparison. Performance is then assessed using a test set of equal size to the training data (400 observations) and the time‐dependent AUC and Brier scores (see Gerds and Kattan 2021). The variables selected by CooPeR lead to consistently better‐performing models across both metrics and causes.

### A.2 Application Example

Figure A.3 shows results of a performance evaluation performed based on a 70%/30% train‐test split of the original bladder cancer data. Models were tuned and fit on the training data, and the remaining 30% of the data were used to assess performance using time‐dependent Brier scores and AUC across 10% quantiles of the event times in the test data. In each case, a cause‐specific Cox model was fit on the variables selected by each of the included methods, such that differences in selected variables are the only relevant factor influencing predictive performance, rather than model‐specific predictive ability.
