# Supplementary material for: High‐Dimensional Variable Selection With Competing Events Using Cooperative Penalized Regression
Source: Biom J. 2025 Feb 18;67(1):e70036. doi: 10.1002/bimj.70036 (PMC11865700; doi:10.1002/bimj.70036)
Supplement: Supplementary file 1 — Supporting Information [file BIMJ-67-e70036-s001.zip › cooper_supplement_v3/results/figures/2-performance-scores.pdf]

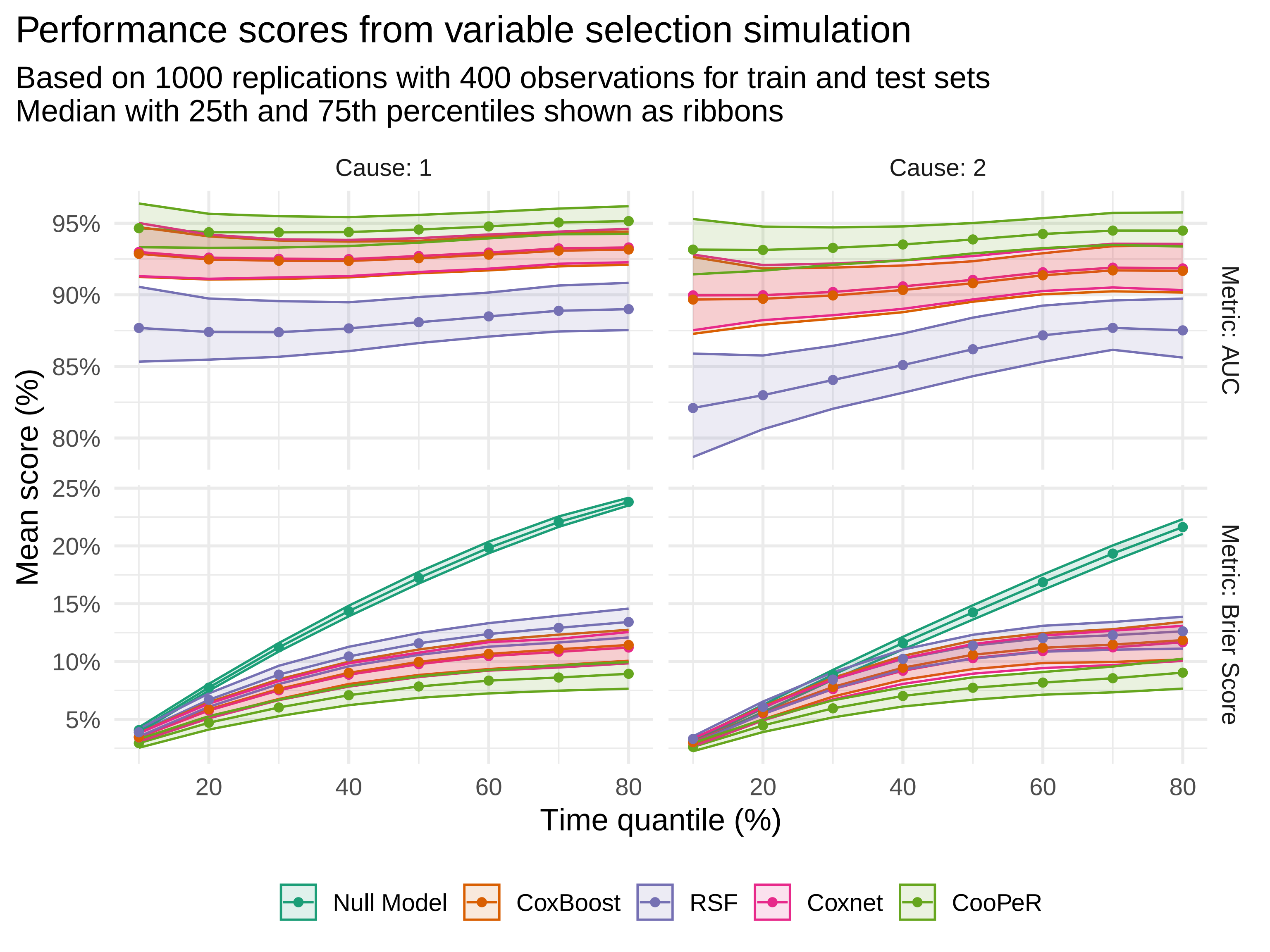
Performance scores from variable selection simulation
Based on 1000 replications with 400 observations for train and test sets
Median with 25th and 75th percentiles shown as ribbons
Cause: 1
Cause: 2
Metric: AUC
Metric: Brier Score
Mean score (%)
95%
90%
85%
80%
25%
20%
15%
10%
5%
20
40
60
80
Time quantile (%)
Null Model
CoxBoost
RSF
Coxnet
CooPeR
