# Supplementary material for: High‐Dimensional Variable Selection With Competing Events Using Cooperative Penalized Regression
Source: Biom J. 2025 Feb 18;67(1):e70036. doi: 10.1002/bimj.70036 (PMC11865700; doi:10.1002/bimj.70036)
Supplement: Supplementary file 1 — Supporting Information [file BIMJ-67-e70036-s001.zip › cooper_supplement_v3/results/figures/3-bladder-performance.pdf]

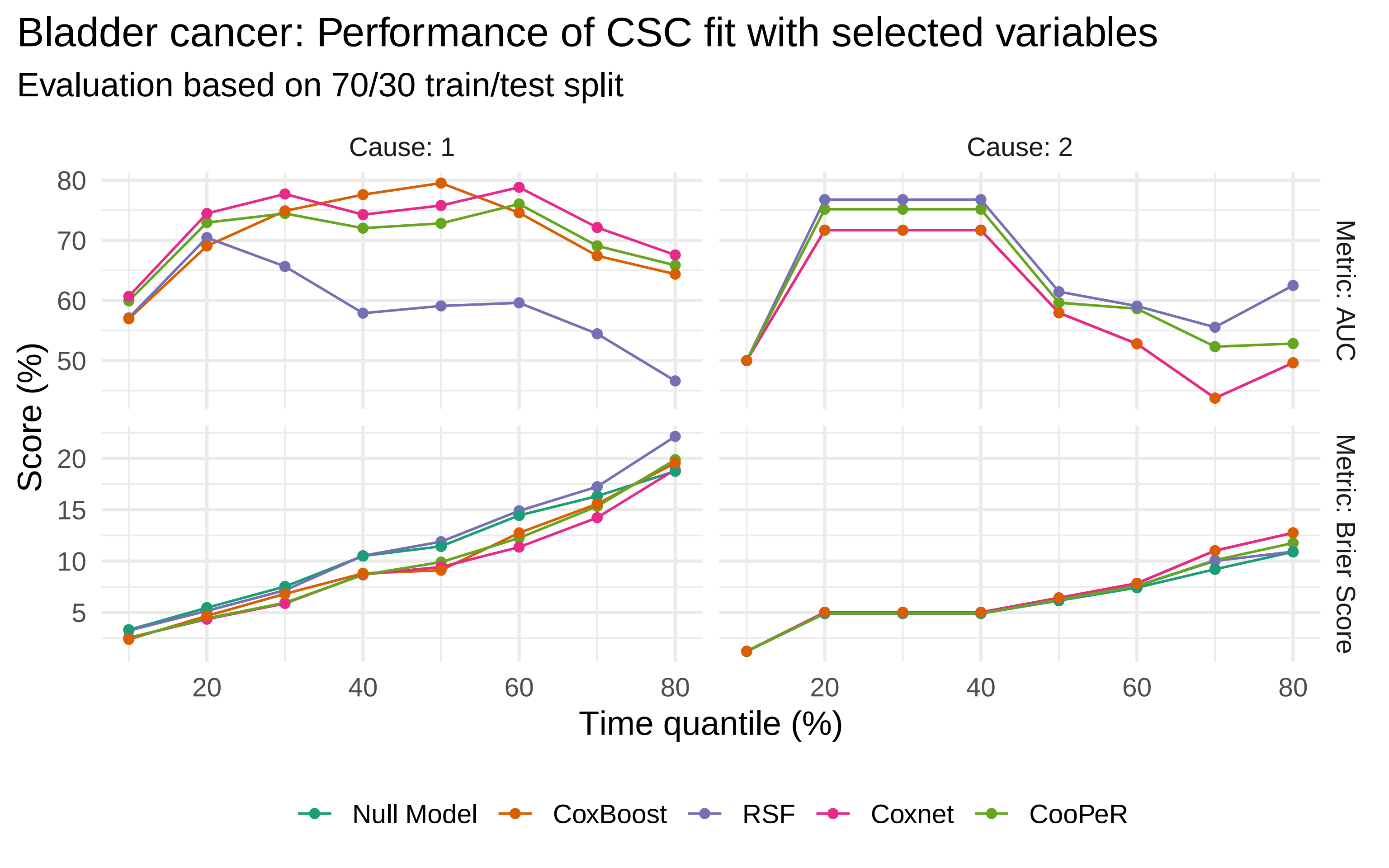

Bladder cancer: Performance of CSC fit with selected variables
Evaluation based on 70/30 train/test split
Cause: 1
Cause: 2
Metric: AUC
Metric: Brier Score
Score (%)
Time quantile (%)
80
70
60
50
20
15
10
5
20
40
60
80
Null Model
CoxBoost
RSF
Coxnet
CooPeR
