# Supplementary figures and images for: High‐Dimensional Variable Selection With Competing Events Using Cooperative Penalized Regression
Source: Biom J. 2025 Feb 18;67(1):e70036. doi: 10.1002/bimj.70036 (PMC11865700; doi:10.1002/bimj.70036)

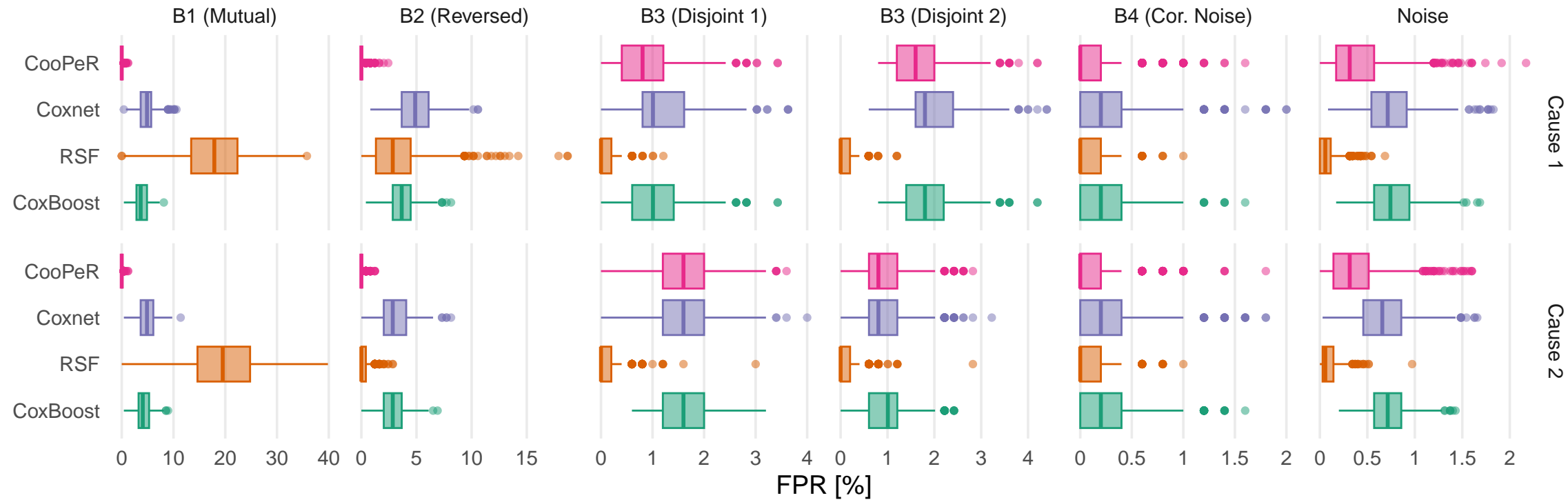

Supplement: Supplementary file 1 — Supporting Information [file BIMJ-67-e70036-s001.zip › cooper_supplement_v3/results/figures/2-fpr-equallambda.pdf]

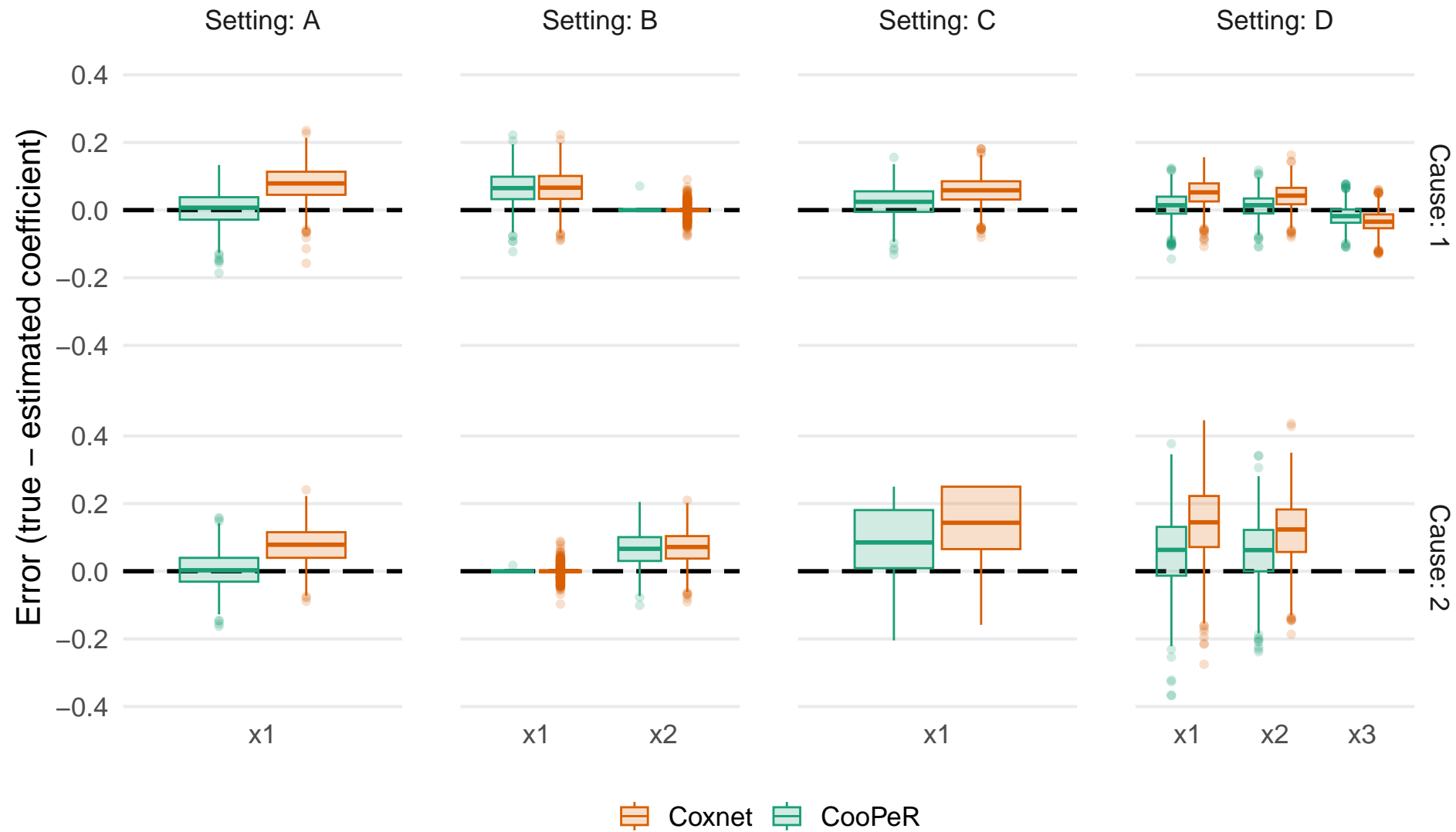

Supplement: Supplementary file 1 — Supporting Information [file BIMJ-67-e70036-s001.zip › cooper_supplement_v3/results/figures/1-poc-boxplot-errors.pdf]

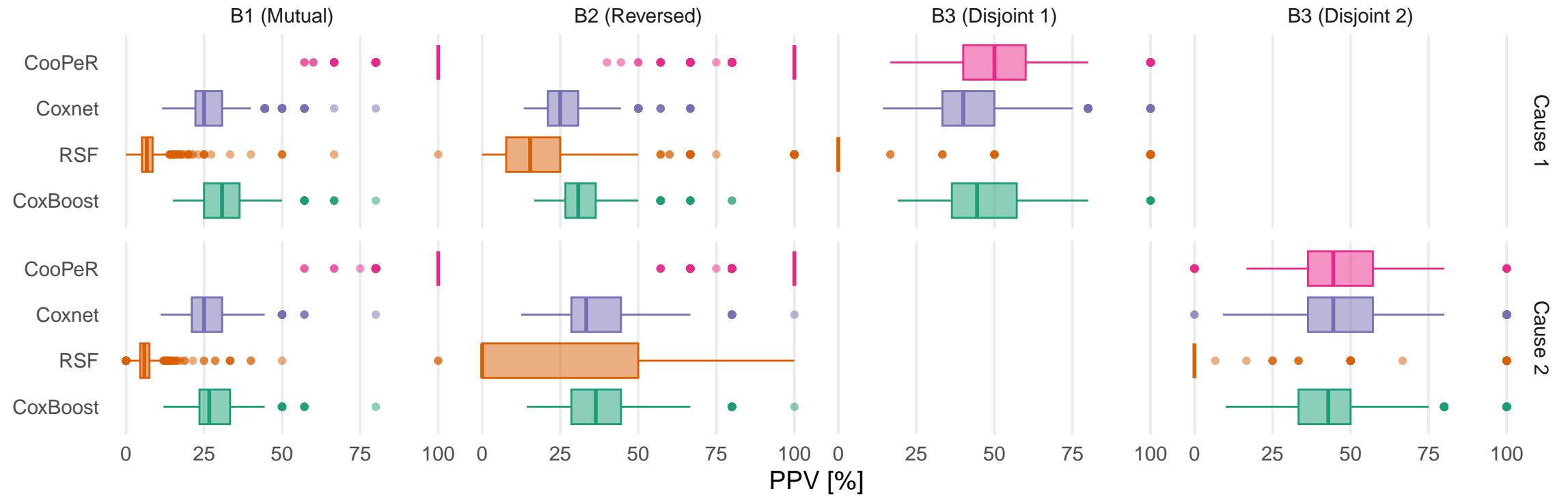

Supplement: Supplementary file 1 — Supporting Information [file BIMJ-67-e70036-s001.zip › cooper_supplement_v3/results/figures/2-ppv-equallambda.pdf]

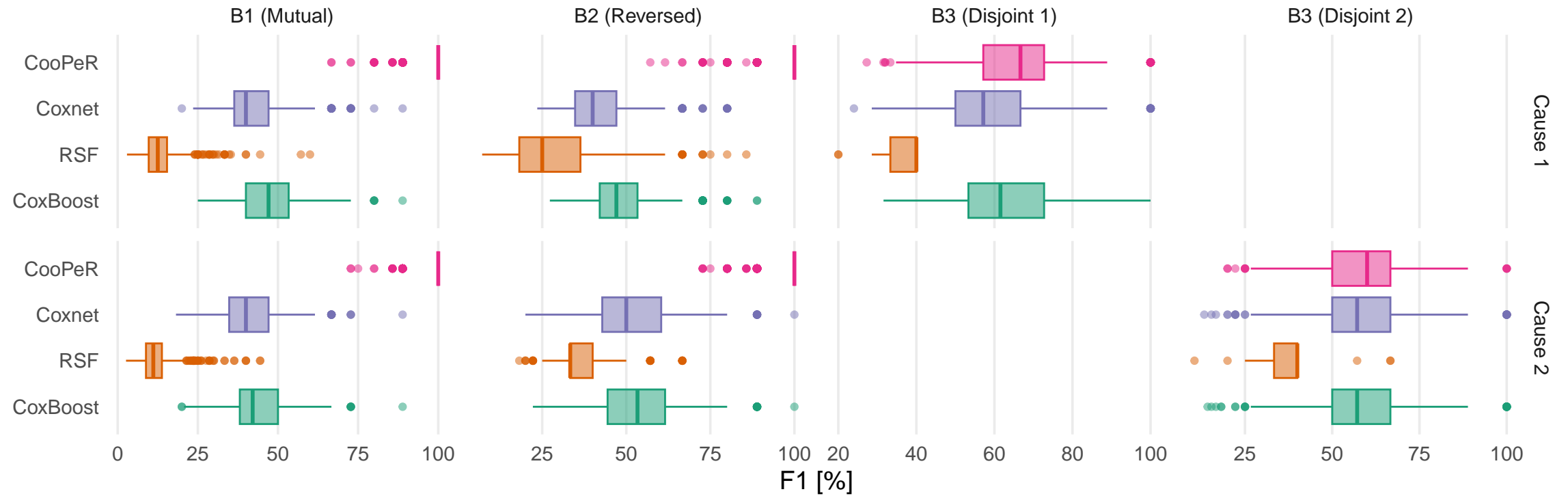

Supplement: Supplementary file 1 — Supporting Information [file BIMJ-67-e70036-s001.zip › cooper_supplement_v3/results/figures/2-f1-equallambda.pdf]
